# Multidomain Cognitive Tele-Neurorehabilitation Training in Long-Term Post-Stroke Patients: An RCT Study

**DOI:** 10.3390/brainsci15020145

**Published:** 2025-01-31

**Authors:** Marianna Contrada, Gennarina Arabia, Martina Vatrano, Caterina Pucci, Isabel Mantia, Federica Scarfone, Giusi Torchia, Maria Quintieri, Antonio Cerasa, Loris Pignolo

**Affiliations:** 1 S. Anna Institute, 88900 Crotone, Italy; marianna.contrada@gmail.com (M.C.); martinavatrano92@gmail.com (M.V.); puccicaterina88@gmail.com (C.P.); isabel.mantia@virgilio.it (I.M.); federica.scarfone10@gmail.com (F.S.); m.quintieri@isakr.it (M.Q.); antonio.cerasa76@gmail.com (A.C.); 2 Institute of Neurology, University Magna Graecia, 88100 Catanzaro, Italy; g.arabia@unicz.it (G.A.); giusi.torchia79@gmail.com (G.T.); 3 IBSBC-CNR, Via T. Campanella, 88100 Catanzaro, Italy

**Keywords:** tele-neurorehabilitation, stroke, multidomain cognitive training, mood, virtual reality rehabilitation system

## Abstract

Purpose: Over the past decade, tele-neurorehabilitation (TNR) has emerged as a vital and effective tool for delivering continuous care to stroke patients, playing a key role in enhancing functional recovery and ensuring consistent access to rehabilitation services. In the field of TNR, various protocols are utilized to ensure effective cognitive stimulation at home. Recent preliminary studies highlight the employment of multidomain cognitive interventions, which would seem to induce more stable and relevant cognitive recovery in stroke patients. A randomized controlled trial (RCT) study was conducted to compare the effectiveness of a TNR multidomain cognitive approach to conventional face-to-face cognitive treatment. Methods: A total of 30 patients with stroke were equally enrolled and randomly assigned to the experimental and control groups. In the experimental group, patients received sessions of home-based cognitive virtual reality rehabilitation system (VRRS) training. The control group underwent traditional face-to-face cognitive multidomain treatment at the hospital. The therapy was given for one hour every day for four weeks in both groups. Specific cognitive domains, including memory, praxis skills, executive functions, and speech therapy, were stimulated in the procedure. Neuropsychological evaluations were performed at three timepoints: at baseline (T0), at the end of TNR (T1), and six months later (T2). Results: The TNR group demonstrated significant improvements in working memory and language abilities, as well as in depressive symptoms and caregiver burden, with an average decrease of 2.07. Most of this improvement persisted 6 months after treatment. The group that received face-to-face cognitive treatment showed improvements (not persisting at T2) after treatment in a task measuring constructive apraxia and alternating attention with the cognitive skill of set-shifting. Conclusions: According to our findings, multidomain cognitive TNR may be useful in enhancing cognitive outcomes in stroke populations (even six months after treatment concludes). TNR may also be a viable way to deliver these interventions since it boosts people’s motivation to train and, consequently, their adherence to treatment while also having a positive effect on caregivers’ distress management.

## 1. Introduction

Stroke patients often experience chronic cognitive dysfunctions, persisting for up to three years post-incident [1,2]. Limited access to appropriate care after hospital discharge can hinder recovery and negatively affect long-term outcomes [3]. Recently, advancements in technology have introduced Tele-NeuroRehabilitation (TNR), which uses telecommunication tools for remote assessment, support, and rehabilitation [4]. As part of telemedicine, TNR offers an effective way to address the growing demand for rehabilitation by providing accessible, cost-efficient care, especially for patients in remote areas. Studies indicate that TNR is as effective as conventional therapy in improving cognitive functions like memory, verbal fluency, and executive skills [5,6,7].

Generally, remote TNR services are focused on specific cognitive deficits. The complex and often extensive nature of post-stroke cognitive impairment means that focusing on domain-specific cognitive outcomes may not fully reflect the interconnected cognitive impairments that arise in stroke [8]. There is more work to be performed to address the narrow focus of some cognitive rehabilitation techniques that focus on a specific domain of cognitive function. However, the variety of cognitive treatments that TNR systems may offer may jeopardize their validity and undoubtedly make it more difficult to extrapolate the findings to other situations [9]. Although one-domain cognitive intervention focuses on highly specific cognitive abilities, the single domain ignores the complex interplay between various mental processes that are necessary to establish and maintain a healthy and viable mental state that can think flexibly enough to engage with the world in productive ways [10]. Recently, our group [11] demonstrated the effectiveness of a multidomain cognitive approach for stroke patients at home.

The aim of this study is to demonstrate the effectiveness of a previously validated multidomain TNR intervention [11] in comparison with a traditional face-to-face cognitive multidomain treatment at a hospital using a randomized controlled trial (RCT) study.

## 2. Material and Methods

### 2.1. Participants

Recruitment and treatment were conducted at the Institute S. Anna of Crotone from January 2022 to September 2024.

Inclusion criteria were as follows: (a) diagnosed with ischemic stroke (middle or anterior cerebral arteries); (b) >18 years old; (c) patients discharged; (d) persistent mild cognitive impairments fulfilling the Peterson criteria for MCI [12]; (e) clinical conditions stable; (f) >8 months from event; (g) no clinical complications incompatible with rehabilitation training; and (h) able to receive in-home NeuroRehabilitation services. Exclusion criteria were as follows: (a) presence of other non-vascular brain lesions; (b) history of psychiatric disorders and/or drug and/or alcohol abuse; (c) severe aphasia; and (d) severe visual deficits, traumatic brain injury, and brain tumor.

Each subject gave written informed consent. The study was approved by the Ethical Committee of the Central Area Regione Calabria (n. 113; 17 April 2018) according to the Helsinki Declaration.

All of the participants had the characteristics outlined below.

### 2.2. Study Design

Our blind, randomized, controlled trial consisted of four main stages. In stage one, patients meeting the inclusion criteria were recruited without being informed of their group assignment or training rationale (clinical center for the control group; home-based for the experimental group). The psychologist, primary researcher, and data entry assistants were blinded to group membership. In stage two, eligible stroke patients underwent neuropsychological baseline evaluation (T0). In stage three, participants were randomized into experimental or control groups using a computer-generated, site-stratified schedule based on Mini-Mental State Examination (MMSE) and gender. Random numbers were assigned, placed in envelopes, and opened to determine group allocation. Different blinded research assistants administered each step of the process. Neuropsychologists and data entry assistants were blinded to all phases of the examination. After that, all participants began receiving 60 min sessions of specialized cognitive therapy for four weeks, lasting an hour every day, five days a week.

1. Tele-NeuroRehabilitation Group (TNRG): underwent home-based Virtual Reality Rehabilitation System (VRRS of the Khymeia group, Noventa Padovana, Italy; https://khymeia.com/it/, accessed on 21 December 2024) training, which included multidomain cognitive TNR, in accordance with our previous study [11]. During the initial TNR phase (technology training, wi-fi connection), caregivers provide support to help the patient in the use of the technology and perform constant monitoring in assisting the person during TNR sessions.
2. Control Group (CG): received face-to-face traditional cognitive treatment, where participants received only face-to-face cognitive conventional rehabilitation. Face-to-face cognitive rehabilitation represents the gold standard practice in cognitive rehabilitation and reflects traditional therapeutic modalities, which are proven to be effective in improving cognitive abilities in patients with neuropsychological disorders [13,14].

Finally, neuropsychological evaluations were further performed at the end of treatment and (T1) and six months later (T2)

### 2.3. Assessment: Clinical, Mood, and Neuropsychological Status

At baseline, we assessed the general cognitive status using the MMSE [15] and the Cognitive Reserve Index questionnaire (CRIq) [15] for entry study purposes.

Next, at all timepoints, a complete neuropsychological assessment was performed using the following: (a) Rey Auditory Verbal Learning Test (RAVLT) [16], the auditory verbal learning test; (b) Digit Span (Verbal and Spatial Immediate Memory Span) [17] to assess verbal short-term memory, defined as the system that allows for temporary storage of information, and is crucial in everyday tasks; (c) Trail-Making Test A-B (TMT A-B) of visual attention and task-switching [18]; (d) Copying drawings without and with programming elements (CD and CDP), consisting of the free hand copy (CD—without programming elements) or with programming elements (CDP—with programming elements) [16]; (e) assessing the ability of naming nouns and verbs (Battery for Analysis of Aphasics Deficit, B.A.D.A.) [19].

Finally, we used the following questionnaires for mood assessment: (a) Beck Depression Inventory II (BDI-II) [20] depression inventory self-report; (b) State-Trait Anxiety Inventory (STAI) [21] to assess state and trait anxiety (X-1; X-2); and (c) Caregiver Burden Inventory (CBI) [22]. Short Form Health Survey-36 (SF−36) [23] is a self-report questionnaire that measures the quality of life in relation to the health of the subject. It is divided into two components: mental component summary (MCS) and physical component summary (PCS).

### 2.4. Multidomain Cognitive Treatment

The experimental group used the VRRS HomeKit, a tablet-based system in a briefcase enabling motor, cognitive, and speech therapy at home. Guided by a therapist via the Tele-Cockpit and supported by a caregiver, the system offers teletraining, telemonitoring, teleconsultation, and diagnostic imaging streaming. Exercises were tailored to patients’ cognitive abilities, with adjustable parameters such as duration, repetitions, and difficulty level, alongside features like gradual progression, acoustic feedback, and optional instructions. Specific exercises targeted memory, attention, and motor skills.

Control group underwent traditional neurocognitive treatment using paper and pencil based and delivered according to the resources offered by the S. Anna Institute. All exercises conform to a task-oriented paradigm. Every patient received the same measure of training. All the pencil-and-paper activities were adapted from work by Iannizzi et al. [24]. The targeted activities were selected to improve language, perception, spatial and temporal orientation, memory, attention, and visual–spatial abilities.

The description of all exercises used in various cognitive domains for the experimental and control groups is shown in Table 1.

### 2.5. Statistical Analysis

Statistical analysis was performed using SPSS 26 software (version 26; Statistical Package for Social Sciences; www.spss.it, accessed on 1 January 2025). Summary statistics are expressed as means and standard deviations. The Shapiro–Wilk normality test was used to examine the distribution of each variable. Non-parametric techniques were chosen for the analysis due to the small sample size (n = 15) and the non-normal distribution of the variables (0.64 ≤ W ≤ 0.87). This approach is considered to provide more accurate results when the sample size is small or when tables are sparse or imbalanced [25,26]. In T0, the comparisons of socio-demographic parameters, neuropsychological tests, and mood assessment between groups were evaluated using the Mann–Whitney test.

The neuropsychological and mood assessments were compared across time points for each group using the Wilcoxon test and between groups in T1 and T2 using the Mann–Whitney test. The level of significance was set at *p* < 0.05. The effect size was calculated as the absolute value of Z/√(N), where Z is the Z-statistic of the statistical test, and N is the total number of subjects. The effect size results were considered as follows: r < 0.1, not significant; 0.1 ≤ r < 0.3, low; 0.3 ≤ r < 0.5, medium; r > 0.5, high.

## 3. Results

**Figure 1:**
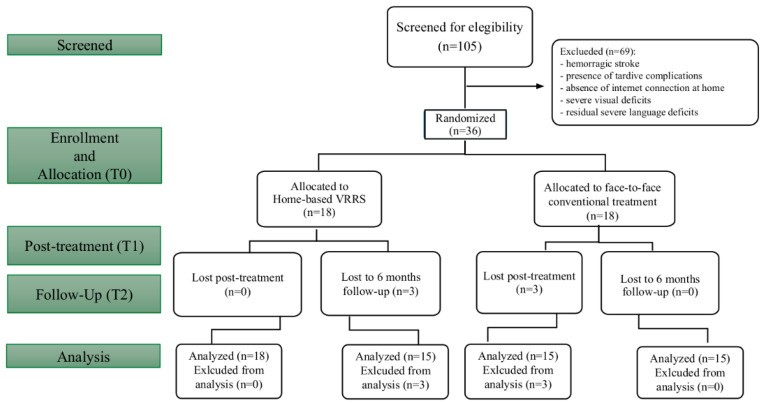
The CONSORT flow diagram illustrates the stages of a parallel randomized trial in which two groups of stroke patients received either traditional (control group) or home-based (experimental group) multidomain cognitive training.

Of the initial cohort, 69 stroke patients were excluded because they did not meet the study’s inclusion criteria. Thirty-six ischemic post-stroke patients were enrolled. Three participants in the TNR and CG groups did not terminate the T2 and T1 phases (Figure 1) because they had a second stroke event. At the time of inclusion, the TNRG and CG were perfectly matched for all demographic and clinical variables (Table 2). Table 3 and Table 4 report the characteristics of every single patient.

In TNRG, B.A.D.A Actions (Z = −2.807, *p* = 0.001, r = 0.51) showed significant differences between T0 and T1, with an increasing trend. However, performance tends to increase in the language domain (B.A.D.A Naming and B.A.D.A Actions; Z = −2.217, *p* = 0.02, r = 0.40 and Z = −1.895, *p* = 0.03, r = 0.35, respectively) and memory domain (Digit Span FW and Digit Span BW; Z = −2.134, *p* = 0.02, r = 0.39 and Z = −1.680, *p* = 0.04, *r* = 0.31, respectively) six months after treatment starts. The mood evaluation revealed decreasing values: the BDI II (Z = −1.855, *p* = 0.03, r = 0.34) and the CBI (Z = −1.684, *p* = 0.04, r = 0.31) both had statistically significant outcomes between T0 and T1.

In contrast, performance in the attention domain worsened between T0 and T1 in the TMT B (Z = −2.040, *p* = 0.02, r = 0.37) and TMT B-A (Z = −2.118, *p* = 0.02, r = 0.39) and between T1 and T2 (Z = −2.191, *p* = 0.01, r = 0.40; Z = −2.395, *p* =0.007, r = 0.44; respectively; for TMT B and TMT B-A) (Table 5 and Table 6).

In the CG, instead, a significant difference between T0 and T1 was found in CD (Z = −1.783, *p* = 0.04, r = 0.33) and CDP (Z = −1.867, *p* = 0.03, r = 0.34). However, in this case, attentional performance improved between T0 and T1 in TMT A (Z = −2.158, *p* = 0.01, r = 0.39) (Table 5 and Table 6).

Similar analyses were conducted for MCS and PCS, but no statistically significant differences were found (Table 5 and Table 6).

Significant differences were observed and compared to TNRG and CG in B-A and B.A.D.A Actions in T1 (Z = −1.884, *p* = 0.03, r = 0.34 and Z = −2.131, *p* = 0.02, r = 0.40, respectively) and in the language domain (B.A.D.A Actions and B.A.D.A Naming; Z = −2.400, *p* = 0.008, r = 0.44 and Z = −2.414, *p* = 0.009, r = 0.44, respectively) in T2.

## 4. Discussion

Long-term post-hospital discharge telerehabilitation programs have generally been shown to be successful in enhancing particular cognitive abilities, like language and memory [11,27,28], but there is a lack of RCT studies [27,29,30,31,32,33]. In this study, we present preliminary findings on the distinctions between a well-known TNR training program and a conventional hospital neurorehabilitation approach. Although those receiving home treatment showed wider improvement, both approaches generally resulted in improved cognitive recovery in long-term stroke patients. In fact, following treatment, patients in the TNR group performed better in the language domain (B.A.D.A, Digit Span), and worse in the attention domain (TMT), while the control group improved in attention (TMT) and visuospatial (CD/CDP) abilities. It is interesting to note that whereas hospitalized patients showed no discernible changes at T2, the great majority of cognitive recovery in the TNR group continued after six months of treatment. Interestingly, TNR patients showed significant improvements in depression and caregiver burden, which did not persist after 6 months. Worsening in the attention domain may be due to the way the exercise is performed and patient fatigue. The better cognitive picture in the TNR group compared to those receiving hospital treatment may be explained by the patients’ improved moods and the decreased caregiver load. In fact, VRRS treatment positively influenced the recovery of health perception and mood. Many studies suggest that RCT is an effective tool to improve motor, cognitive, and mood outcomes in post-stroke patients. This demonstrates that treatment stimulates patient motivation and promotes continuity of care [5,27,34,35,36].

Family caregivers offer stroke survivors informal, unpaid care after they are released from the hospital. A survey found that 82% of family caregivers spent more than eight hours a day caring for stroke victims [37] and that stroke caregivers’ health steadily deteriorated within a year following a stroke [38]. A lot of systematic reviews and meta-analytic studies demonstrated that telerehabilitation assistance has a positive effect on the caregivers’ burden and distress management, reducing distress and encouraging positive aspects of caring [5,39,40]. In the opposite direction, it has been repeatedly demonstrated that higher caregiver burden correlates with worse patient outcomes [41,42]. For this reason, it is reasonable to hypothesize that the better cognitive outcome detected in the stroke groups at home could be determined by the lower burden detected in the caregivers. Further studies using more advanced statistical models (i.e., structural equation modeling) are warranted to confirm this suggestion.

The presence of a low beneficial effect of multidomain cognitive training in the control group could be explained by the reduced effectiveness of therapy in the long-term period. Indeed, according to the most recent international estimates, 60–70% of people who suffer a stroke experience cognitive deficits during the acute phases of recovery [43]. The time window considered in almost all these studies is within 6–12 months after their stroke. It is still unknown how rehabilitation affects post-stroke recovery and how much it depends on the patient’s chronicity [44]. The idea of a proportional recovery rule with a “critical window for recovery” during the first three to six months after a stroke has been largely recognized in the field [45]. However, a large number of studies have been performed considering the motor domain, whereas there is a paucity of evidence about the critical windows for cognitive recovery. We found that even in late chronic stages, improvements in some cognitive functions were found, although they did not persist for long periods of time.

In addition, despite the limitations of technology in some rural areas, such as limited access to internet connections, environmental disturbances, and cultural variations, TNR can overcome these challenges in the clinical setting with caregivers’ support and active involvement. Caregivers play a crucial role in mitigating technological barriers and enhancing the patient’s focus during cognitive treatment sessions, making them essential for overcoming obstacles and maximizing the benefits of cognitive telerehabilitation [27]. These complexities require continuous challenges to adapt technology to the available resources of post-stroke patients, taking into account the cost-effectiveness ratio. TNR, in fact, eliminates the stress and time associated with transporting patients to hospital services—especially for those living far from major facilities—while ensuring home access to physical and cognitive training, thereby reducing costs and healthcare expenses [27,35,46,47].

## 5. Limitations

The main limitation of this study is its limited sample size, which prevented us from directly comparing groups using statistical parametric techniques. It is crucial to emphasize how challenging it is to enroll long-term patients in neurorehabilitation treatment, whether at home or in a hospital. In fact, only 30 of the 105 patients in the initial cohort were enrolled and completed the entire course of treatment. To more effectively get over this inherent drawback of the monocentric method, future multicentric research is necessary. Our study should be considered as a pilot, with promising future applications to overcome the barriers related to access to services caused by distance or difficulty of a patient’s mobility.

## 6. Conclusions

In recent years, the potential usefulness of cognitive training in normal aging and in patients with mild to moderate cognitive impairment after stroke has received increasing attention [27,48]. Our results suggest that multidomain cognitive TNR could be effective in improving cognitive outcomes in populations with ischemic stroke (even six months after the end of treatment) and that Virtual Reality could represent a promising means to administer such interventions, as it increases individuals‘ motivation to train and thus their compliance to treatment, with a beneficial impact on caregivers’ management of distress.

## Figures and Tables

**Table 1:** Multidomain cognitive treatment in experimental and control groups.

	TNRG		CG		
Main Domain	Task	Description	Task	Description	Task Duration
Logical/Logical-mathematical skills	Logical associations–images/words	Images/words appear on the screen to be matched according to a logical relationship	Proverbs	The assignment is to elucidate the meaning of the proverb	
	Find the extraneous word/image	The subject must find the unrelated word/image	Cruci-number	The task requires to find for each letter indicating the result of the corresponding operation and place it in the grid by placing only the digit per box horizontally or vertically	10′
	Calculate total price/ rest	The subject must figure out how much needs to be paid in total or how much change is owed based on the information displayed on the screen	Finds the mistake	From a list of comparable terms, the subject is asked to delete the intrusing word	
Spatial perception/praxis skills	Puzzle	To create an accurate and whole jigsaw puzzle, the subject must rearrange a collection of jumbled jigsaw pieces	How much is it?	An object is illustrated and asked for an estimate of the price	
	Drawing by neglect	An incomplete figure appears on the screen on one side to be completed by the patient	Copy of drawings	A few drawings are given to the patient to copy	10′
	Rotation	Objects with different rotations appear on the screen. To finish a sequence, the patient has to identify which rotation is accurate	Spontaneous drawing	A spontaneous figure drawing exercise is given to the patient	
Attention	Attentional matrices	A sheet with one or more matrices (stimulus/target) to be crossed in a grid with many distractions will show up on the screen	How many are?	The participant is exposed to target stimuli. The subject must recognize and classify each target stimulus on the sheet after they have been recognized and given names	
	Recognize/match banknotes/coins	An overview screen will be presented with a series of random banknotes or coins in disarray. The task will be to recognize or match the front or back banknotes or coins	Seek the target stimulus	The patient has to search for the target stimulus among many distracting stimuli	10′
	Find differences	The subject will have to find the differences between two apparently identical images	Crucipuzzle	A random pattern of letters is displayed. Using either a vertical or horizontal search, the patient must locate the hidden words	
Executive functions	Planning	Snatches of a brief story are presented on screen in a random order. The participant has to put them back in chronological order	Beating hands and/or feet	A series of words and numbers are read out, the subject must clap their hands when they hear a word and stomp their feet when they hear a number; or clap their hands when the subject hears the name of a fruit and stomp their feet when the name of an animal is read out.	
	Change color/shape/dimension/all	The subject is asked to choose from a set of figures a geometric figure that differs from the target simply in terms of shape, only in terms of color; only in terms of size; or in terms of color, shape, and dimension altogether	Go no-go	The patient will be given contradicting instructions, such as lists of colors and tree names that will be read out, with the patient being required to clap his hands when he hears the name of a color and to remain still when he hears the name of a tree.	10′
	Collect money up	A set of coins (starting with cents) or a set of banknotes (starting with EUR 5) appear on the screen. The subject is asked to collect the indicated amount	Planning	Cards with randomly arranged sentences that comprise snippets of a brief narrative will either be read aloud or given to the subject. After that, the participant will be required to rearrange them in a chronological order.	
Memory	Open safe (backward/forward)	A closed safe will appear on screen, and a sequence of numbers to be memorized will be shown. After a few seconds, the numbers will disappear, and to open the safe, the subject has to put the sequence in the same order or backward	Shopping list	A list of words that includes, for example, “food”, must be read to the patient by the rehabilitator. The patient will need to commit it to memory	10′
	Visual memory	On the screen, pairs of cards (geometric shapes or animals) will be presented for the person to memorize. Then, the cards will turn over and the person will have to remember the position of the pairs	Memory cards	Pairs of cards (representing animals, foods, etc.) will be presented for the person to memorize. Then, the cards will turn over and the person will have to remember the position of the pairs	
	Word memorization	A list of words that show up on the screen must be committed to memory by the user. These terms will then vanish and turn up in a list of distracting words	Sequential image memory	A set of figures is shown to the patient for memorization	
Language	Identify the action	The subject must identify the action illustrated on screen	Fluency	All words that start with particular syllables or fall in a specific category (such as foods, colors, etc.) should be listed either orally or in writing	10′
	Reconstruct the word	Letters appear on screen that the participant must utilize to piece together the correct word	Denomination	The subject is asked to name the images presented	
	Separate by semantic group	The task requires the subject to sort things into groups based on the semantic categories to which they belong			

**Table 2:** Demographic and clinical data at admission (T0) to multidomain cognitive training.

	TNRG	CG	*p* Value
	Median [Q1–Q3]		
N°	15	15	
Gender n (%) Man	6 (40%)	8 (53%)	0.46
Age	63 [50–69]	70 [65–74]	0.09
Education	8 [5–13]	8 [7.25–11.50]	0.48
Days since the event	491 [368–777]	284 [252–408]	0.06
MMSE	23 [22,23]	23 [21–23]	0.42
CRIq Total	92 [83–100]	90 [82–97]	0.50
CRIq Education	88 [84–114]	95 [88–99]	0.32
CRIq Work	90 [88–110]	96 [86.75–112.25]	0.31
CRIq Leisure Time	87 [79–102]	82.50 [76.50–94.5]	0.22

Q1 (first quartile); Q3 (third quartile); MMSE (Mini Mental State Examination); CRIq (Cognitive Reserve Index questionnaire).

**Table 3:** CG Characteristics.

CG Subject	Gender	Age	Diagnosis	Time from Event (Days)	MMSE	CRIq Total
1	F	75	Left fronto-parietal stroke	275	23.3	97
2	F	72	Fronto-insular ischemic stroke	326	23.3	72
3	F	65	Left capsular stroke	252	23.2	102
4	M	62	Bilateral frontal stroke	284	23	90
5	F	65	Right paraventricular ischemic stroke	1095	23.09	70
6	F	51	Right temporal fronto-parietal stroke	646	23.2	93
7	M	72	Right temporal fronto-parietal stroke	247	23	84
8	M	74	Right temporo-parietal stroke	252	23.3	126
9	F	57	Right parietal occipito-temporal cortico-subcortical stroke	408	22.97	88
10	F	57	Left temporal stroke	251	23	82
11	M	70	Left paramedian stroke	273	23.4	94
12	M	71	Right frontal subcortical stroke	248	22.7	120
13	M	82	Right paramedian ponto-mesencephalic stroke	404	21.31	85
14	M	65	Left cerebellar stroke	1168	23	93
15	M	75	Left temporo-insular stroke	300	13	79

**Table 4:** TNRG Characteristics.

TNRG Subject	Gender	Age	Diagnosis	Time from Event (Days)	MMSE	CRIq Total
1	M	65	Left fronto-temporal stroke	734	23	83
2	M	69	Left fronto-temporal stroke	491	18.27	78
3	F	63	Left parieto-temporal stoke	420	22.27	68
4	M	62	Bilateral frontal stroke	1220	23	92
5	F	76	Right frontoparietal stroke	384	23.30	124
6	F	67	Right insulo-temporo-parieto-frontal stroke	914	23	143
7	F	47	Right thalamic stroke	1038	23	77
8	M	49	Right occipito-parietal stroke	246	23	100
9	M	53	Right parietal stroke	511	23	84
10	M	68	Left temporal stroke	249	23	83
11	F	62	Right temporal fronto-parietal stroke	777	23	129
12	M	49	Tail stroke of the right ventricle nucleus	755	23	93
13	F	75	Left cerebellar stroke	368	23	91
14	F	50	Right temporal fronto-parietal stroke	384	23.2	93
15	F	75	Right cerebellar stroke	243	23	127

**Table 5:** Descriptive statistics results for neuropsychological and mood assessment in TNRG and CG in T0, T1, and T2.

	T0		T1		T2		Cut-Off
	TNRG	CG	TNRG	CG	TNRG	CG	
Test	Median [Q1–Q3]						
**MEMORY**							
RAVLT	40.2 [30.5–51]	48.15 [31.05–51.2]	36.4	42.55	44.1	43.95	>28.52
			[32.2–44]	[35.89–50.6]	[27.78–51.88]	[28.47–50.97]	
RAVLT—Retrieval	9.5	10.8	9.9	10.58	9.7	9.5	>4.68
	[6.40–12.30]	[5.25–13]	[7.8–11.8]	[5.28–11.25]	[5.05–12.2]	[4.44–12.75]	
Digit Span FW	4.13	5.12	4.39	4.78	5.28	4.98	>4.26
	[3.75–5.13]	[3.87–5.39]	[3.78–5.2]	[4.23–5.58]	[3.81–5.99]	[3.93–5.46]	
Digit Span BW	3	2.78	2.99	3.34	3.08	3.06	>2.65
	[1.96–4.21]	[2–3.50]	[2.08–4.09]	[2.26–3.95]	[2.86–4.4]	[2.52–3.53]	
**VISUO-SPATIAL ABILITIES**							
CD	9.4	7.65	10.6	10.2	10.1	9.8	>7.18
	[7.1–11.4]	[6.35–9.83]	[9.25–11.40]	[6.33–10.68]	[8.4–11.1]	[6.21–10.4]	
CDP	68.8	56.28	68.4	64.25	57.1	61.9	>61.85
	[65.5–70]	[18.28–68.58]	[59.30–69.50]	[41.60–70.18]	[56.1–70]	[44.2–69.68]	
**ATTENTIONAL AND EXECUTIVE FUNCTIONS**							
TMT A (msec)	55	80	58.49	56	67.5	64.3	<94
	[50–121]	[55–186.57]	[46–98.25]	[41.81–178.67]	[39.1–115.82]	[42–99]	
TMT B	154.97	179.5	227.5	156	186.25	216.75	<283
(msec)	[109.18–227]	[110–479.49]	[167–329.59]	[103.75–354.22]	[119.63–392.08]	−123.32	
TMT B-A	70.07	118	183.35	84.5	87	129	<187
(msec)	[49.5–141.08]	[20.75–195.18]	[104.25–276.19]	[48.75–196.12]	[46.91–233.99]	[53.5–258.42]	
**LANGUAGE**							
B.A.D.A. Naming	27	27	29	28	29	28	
	[26–30]	[25–28.25]	[26.75–30]	[26–28.25]	[28–30]	[26.25–28.75]	
B.A.D.A. Actions	24	23.5	28	25.5	26	23	
	[22–27]	[21.75–25.25]	[25–28.25]	[21.75–28]	[25.5–28]	[20–26]	
**MOOD**							
BDI II	11	12	10	11	20	12	>13
	[7–26]	[5–19]	[3–20]	[5.75–18]	[9.5–28.5]	[8–18]	
STAI X-I	41	44	42	38	42	40	>40
	[37–48]	[36.5–49]	[35–54]	[35–47.5]	[34.5–47.25]	[34–46.25]	
STAI X-II	48	41	44	41	48.5	44	>40
	[39–52]	[35.5–52]	[35–55]	[37–45.5]	[33.75–55.75]	[34.25–50.5]	
CBI	23.5	27.5	20.5	19	23	21	
	[17.75–37.5]	[14.5–37.75]	[16.75–31]	[14–35.5]	[11–37.75]	[12.5–34.25]	
**QUALITY OF LIFE**							
MCS	48.75	31.88	39.38	39.88	55.98	50.82	
	[36.5–58.19]	[21.82–60.67]	[35.06–61.25]	[23.78–64.4]	[43.13–78.31]	[33.04–58.45]	
PCS	41.25	26.25	43.13	37.5	42.82	31.57	
	[24.69–50.31]	[15–58.75]	[27.82–57.51]	[15.32–50]	[29.85–62.82]	[15.31–55.78]	

Q1 (first quartile); Q3 (third quartile).

**Table 6:** Results for neuropsychological and mood assessment in TNRG and CG in T0, T1, and T2.

TEST	TNRG T0 vs. T1 *p* value	TNRG T1 vs. T2 *p* value	TNRG T0 vs. T2 *p* value	CG T0 vs. T1 *p* value	CG T1 vs. T2 *p* value	CG T0 vs. T2 *p* value
**COGNITIVE**						
RAVLT	0.13	0.09	0.46	0.41	0.27	0.35
RAVLT—Retrieval	0.31	0.51	0.41	0.19	0.33	0.06
Digit Span FW	0.18	0.05	0.02 *	0.16	0.46	0.19
Digit Span BW	0.43	0.23	0.04 *	0.05	0.46	0.06
TMT A	0.18	0.47	0.25	0.01 *	0.35	0.07
TMT B	0.02 *	0.01 *	0.19	0.08	0.32	0.31
TMT B-A	0.02	0.007 *	0.37	0.36	0.37	0.21
B.A.D.A.—Objects naming	0.19	0.5	0.02 *	0.13	0.30	0.24
B.A.D.A.—Actions naming	0.001 *	0.12	0.03 *	0.08	0.11	0.22
CD	0.25	0.43	0.06	0.04 *	0.34	0.09
CDP	0.19	0.29	0.22	0.03 *	0.21	0.25
**MOOD**						
BDI II	0.03*	0.05	0.23	0.34	0.29	0.33
STAI X-I	0.47	0.14	0.43	0.40	0.42	0.47
STAI X-II	0.48	0.30	0.44	0.31	0.30	0.36
CBI	0.04*	0.33	0.19	0.20	0.09	0.39
**QUALITY OF LIFE**						
PCS	0.26	0.40	0.08	0.42	0.52	0.49
MCS	0.31	0.06	0.08	0.18	0.48	0.23

Copying of drawing test without programming elements (CD); copying of drawings test with programming elements (CDP); Digit Span ((Verbal and Spatial Immediate Memory Span) direct span; forward, FW, or in reverse span, backward, BW); Trail Making Test A-B (TMT A-B); object naming and action naming, (Battery for Analysis of Aphasics Deficit, B.A.D.A.); Rey Auditory Verbal Learning Test (RAVLT); Beck Depression Inventory II (BDI-II); State-Trait Anxiety Inventory (STAI); Caregiver Burden Inventory (CBI); Mental Component Summary (MCS) and Physical Component Summary (PCS). ‘*’ indicates statistically significant values.

## Data Availability

The raw data supporting the conclusions of this article will be made available by the authors on request due to due to privacy and ethical restrictions.
